# Histopathological Growth Pattern, Proteolysis and Angiogenesis in Chemonaive Patients Resected for Multiple Colorectal Liver Metastases

**DOI:** 10.1155/2012/907971

**Published:** 2012-08-02

**Authors:** Rikke Løvendahl Eefsen, Gert G. Van den Eynden, Gunilla Høyer-Hansen, Pnina Brodt, Ole Didrik Laerum, Peter B. Vermeulen, Ib Jarle Christensen, André Wettergren, Birgitte Federspiel, Gro L. Willemoe, Ben Vainer, Kell Østerlind, Martin Illemann

**Affiliations:** ^1^Department of Oncology, Rigshospitalet, Copenhagen University Hospital, 2100 Copenhagen, Denmark; ^2^The Finsen Laboratory, Rigshospitalet, Copenhagen University Hospital, 2200 Copenhagen, Denmark; ^3^Biotech Research and Innovation Centre (BRIC), Copenhagen University, 2200 Copenhagen, Denmark; ^4^Translational Cancer Research Unit, GZA Hospitals St.-Augustinus, 2610, Antwerp, Belgium; ^5^Departments of Surgery, Medicine and Oncology, McGill University Health Center, McGill University, Montreal, Quebec, Canada H3A 1A1; ^6^Department of Pathology, The Gade Institute, Haukeland Hospital, 5021 Bergen, Norway; ^7^Department of Surgery, Rigshospitalet, Copenhagen University Hospital, 2100 Copenhagen, Denmark; ^8^Department of Pathology, Rigshospitalet, Copenhagen University Hospital, 2100 Copenhagen, Denmark

## Abstract

The purpose of this study was to characterise growth patterns, proteolysis, and angiogenesis in colorectal liver metastases from chemonaive patients with multiple liver metastases. Twenty-four patients were included in the study, resected for a median of 2.6 metastases. The growth pattern distribution was 25.8% desmoplastic, 33.9% pushing, and 21% replacement. In 20 patients, identical growth patterns were detected in all metastases, but in 8 of these patients, a second growth pattern was also present in one or two of the metastases. In the remaining 4 patients, no general growth pattern was observed, although none of the liver metastases included more than two growth patterns. Overall, a mixed growth pattern was demonstrated in 19.3% of the liver metastases. Compared to metastases with pushing, those with desmoplastic growth pattern had a significantly up-regulated expression of urokinase-type plasminogen activator receptor (*P* = 0.0008). Angiogenesis was most pronounced in metastases with a pushing growth pattern in comparison to those with desmoplastic (*P* = 0.0007) and replacement growth pattern (*P* = 0.021). Although a minor fraction of the patients harboured metastases with different growth patterns, we observed a tendency toward growth pattern uniformity in the liver metastases arising in the same patient. The result suggests that the growth pattern of liver metastases is not a random phenomenon.

## 1. Introduction

Worldwide colorectal cancer (CRC) accounts for 1.2 million new cases per year, and CRC is the third most prevalent cause of cancer-specific death in both genders [1]. At the time of diagnosis, 25% of the patients have CRC liver metastases (CRLMs) and additional 50% of patients without initial liver metastases will develop liver metastases during follow-up [2–4]. Untreated, these patients only survive for a few months [4], while chemotherapy and targeted therapy with humanised monoclonal antibody (mAb) against the vascular endothelial growth factor (VEGF), bevacizumab, have prolonged the median survival to about 20 months [5]. The epidermal growth factor receptor (EGFR) inhibitor, cetuximab, in combination with chemotherapy has prolonged median survival with 30–33 months [6, 7]. In 20% of the patients, however, a curative resection of the liver metastases can be achieved [8], increasing the 5-year survival to 30–58% [3, 9], and neoadjuvant chemotherapy allows more patients to have a CRLM resection with curative intent [10, 11]. Today, a combination with capecitabine/5-fluorouracil (5-FU), oxaliplatin, and bevacizumab is a common choice of first line treatment, if the tumour is *Kras* mutated. This emphasises the importance of identification of new predictive markers of response to biological treatment.

**Figure 1:**
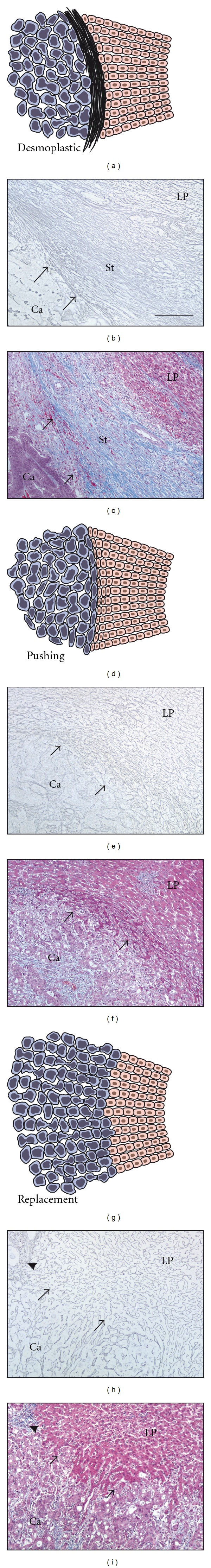
Gordon-Sweet's reticulin and Gomori trichrome stainings of liver metastases. The growth patterns observed in liver metastases are represented by a cartoon (a), (d), and (g). (a): desmoplastic growth pattern, (d): pushing growth pattern, (g): replacement growth pattern. Sections from a patient with a desmoplastic growth pattern are represented in (b) by Gordon-Sweet's reticulin staining and in (c) by Gomori trichrome staining. The desmoplastic stroma is visualised as black lines in (b). In Gomori trichrome staining, collagen turns blue, which is visualised in (c), in the collagen rich desmoplastic stroma (St). The cancer cells (Ca) are red with black nuclei and the liver parenchyma (LP) is visualised by the red cytoplasma stain. Portal tracts, in the upper right corner contains collagen rich tissue, and is blue. Sections from a patient with a pushing growth pattern are represented in (e) by Gordon-Sweet's reticulin staining and in (f) by Gomori trichrome staining. It can be observed that no collagen rich stroma is present at the tumour periphery (black arrows) for liver metastases with a pushing growth pattern. Sections from a patient with a replacement growth pattern are represented in (h) by Gordon-Sweet's reticulin staining and in (i) by Gomori trichrome staining. Like in pushing growth pattern, also replacement growth pattern has no desmoplastic stroma at the invasive front (black arrows). The portal tract, rich in collagen is blue (black arrowhead). In (h), it is hard to tell where the tumour periphery is, but when looking at (i), it is obvious where the cancer cells (Ca) and liver parenchyma (LP) meet. Bar: 200 *μ*m.

Vermeulen et al. described three distinctive morphological growth patterns in CRLM [12]: a *desmoplastic* growth pattern, where the metastases are separated from the liver parenchyma by a rim of connective tissue, preventing direct contact between tumour cells and hepatocytes; a *pushing* growth pattern, where liver cell plates at the liver-parenchyma interface are compressed; a *replacement* growth pattern, where tumour cells infiltrate the liver cell plates, replacing the hepatocytes (Figure 1) [12]. These different growth patterns are characterised by differences in angiogenesis [12, 13]. In the replacement growth pattern, cancer cells expand without eliciting much angiogenesis at the tumour-liver boundary, co-opting the sinusoidal blood vessels between the liver cell plates. The metastases with a pushing growth pattern expand with high angiogenic activity, whereas a lower angiogenic activity has been observed in metastases with a desmoplastic growth pattern [12, 13]. 

The different metastatic growth patterns are also characterised by diversity in expression of proteases and related molecules involved in the breakdown and remodelling of the extracellular matrix processes, which are crucial for cancer invasion and metastasis [14, 15]. The expression pattern of the urokinase-type plasminogen activator (uPA), its cellular receptor (uPAR), and its inhibitor, plasminogen activator inhibitor (PAI-1), has previously been described for CRLM with desmoplastic and pushing growth patterns [14] as well as for primary colon tumours [16, 17]. In liver metastases of the desmoplastic growth pattern, stromal cells at the periphery of the metastases intensively express uPAR, uPA, and PAI-1. In contrast, uPAR and uPA in the pushing growth pattern were only present in necrotic areas within the liver metastases, whereas PAI-1 was found primarily in hepatocytes and in a few myofibroblasts located within the space of Disse [14]. These differences indicate that metastases with different growth patterns differ in their invasive potential. It is still unknown whether the tumour cells, the microenvironment in the liver or other factors determine the growth pattern or the angiogenesis dependency. 

Paku and Lapis recognised two different types of liver metastases in an experimental liver metastasis model [18]. Morphologically, these two distinct growth patterns were believed to be related to the microvascular route of entry and their angiogenic patterns, that is, a sinusoidal-type metastasis and a portal-type metastasis. These morphological patterns were studied in another murine colon carcinoma model [19], demonstrating an increase of endogenous endostatin upon colonisation and growth of colon cancer cells in the murine liver. When the mice were treated with a recombinant endostatin analogue, the tumour growth was reduced exclusively in metastases of the sinusoidal-type. Furthermore, a significant decrease in the number of capillaries per unit tissue area determined by CD31 expression was selectively demonstrated in the sinusoidal-type metastases treated with recombinant endostatin [19]. 

In a recent study, the prognostic value of the growth patterns was evaluated [20]. In the study, it was observed that at 2 years of follow-up after hepatic resection for CRLM, a growth pattern component of pushing was an independent predictor of poor survival [20].

Given the potential clinical relevance of growth patterns and their impact on therapy, the objective of this study was to assess whether the metastases in cases of multiple CRLM had identical growth patterns. If the three former described liver metastases growth patterns represent different underlying patho-biology, a new tool for the stratification of patients for personalised treatment may be given. We analysed liver metastases from chemonaive patients with CRC to avoid the potential effects of prior chemotherapy in combination with or without either bevacizumab or cetuximab.

## 2. Material and Methods

### 2.1. Patient Material

Archival formalin-fixed and paraffin-embedded tissue blocks from 62 liver metastases obtained from 24 chemonaive CRC patients were included in this study. The patients were operated at Rigshospitalet between 2007 and 2010. There were 8 females and 16 males with the age of 43–82 years at the time of diagnosis of the primary tumour. All patients were candidates for resection of their liver metastases without initial downstaging chemotherapy or targeted therapy. Seventeen of the 24 patients had 2 metastases, 3 patients had 3 metastases, 2 patients had 4 metastases, 1 patient had 5 metastases, and 1 patient had 6 metastases (Table 1). After resection, the metastatic tissue had been fixed in formalin for at least 48 hours and thereafter embedded in paraffin. At the time of diagnosis of the primary tumour, 17 patients had synchronous liver metastases. Another 4 patients developed liver metastases within 3 months. Synchronous liver metastases were defined as liver metastases at the time of diagnosis or within three months after diagnosis of the primary tumour. Characteristics of the primary tumour were recorded in the pathology reports from each patient and different features of the primary tumours are listed in Table 2.

Three *μ*m sections were stained with Gordon-Sweet's reticulin staining, and with haematoxylin and eosin according to standard procedures [14]. Two sections of liver metastases of each of the three growth patterns were also stained with the Gomori trichrome staining. The study was approved by the Regional Scientific Ethics Committee (H-2-2011-045) and the Danish Data Protection Agency (2010-41-5623).

### 2.2. Antibodies

A polyclonal antibody (pAb) against uPAR [21] was used. Monoclonal antibodies (mAbs) against human CD31 (clone JC70A), pan-cytokeratin (clone AE1/AE3), CK20 (clone K_s_20.8), CD68 (clone PG-M1), and Ki67 (clone MIB-1), as well as EnVision horseradish peroxidase Mouse (K4001), EnVision horseradish peroxidase Rabbit (K4003) secondary antibodies and an Envision G∣2 Double System Kit (K5361) were purchased from Dako (Glostrup, Denmark) (Table 3). 

### 2.3. Immunohistochemistry

Paraffin sections of 3 *μ*m were mounted on glass slides and deparaffinised with xylene and hydrated through ethanol/water solutions. Antigen retrieval was performed by either pretreatment with protease K digestion (5 *μ*g/*μ*L) at 37°C, or TEG (10 mM Tris, 0.5 mM EGTA, pH 9.0) buffer at 98°C in a T/T Micromed microwave processor (Milestone, Sorisol, Italy). Section pretreatment is listed in Table 3. Sections were blocked for endogenous peroxidase activity by incubation in 1% hydrogen peroxide (H_2_O_2_) for 15 min. The sections were washed in Tris-buffered saline (50 mM Tris, 150 mM NaCl, pH 7.6) containing 0.5% Triton X-100 (TBS-T) and then manually mounted on Shandon racks with immunostaining cover plates (Thermo Shandon, Pittsburgh, PA, USA) for further incubations. Sections were incubated with the primary antibodies overnight at 4°C using EnVision reagents. Each incubation step was followed by washes in TBS-T. The sections were developed with NovaRed (Vector Laboratories SK-4800, Burlingame, CA,) for 9 min and counterstained in Mayer's haematoxylin for 30 sec.

### 2.4. Double Immunostaining

Paraffin sections of 3 *μ*m were double stained with Ki67 and CD31. Staining was performed with the Envision G∣2 Double System kit using the protocol provided by the manufacturer. Antigen retrieval for Ki67 and CD31 was performed in TEG buffer for 20 min at 98°C.

After pretreatment, slides were mounted on Shandon racks as previously described. Subsequently, the endogenous peroxidase activity was blocked by incubation with H_2_O_2_ for 15 minutes. The initial primary antibody reacting with Ki67 was added on the slides and incubated for 2 hours at room temperature. The detection was done with a secondary antibody linked to 3, 3′-diaminobenzidine (DAB). Subsequently, the second primary antibody, reacting with CD31, was added to the slides and incubated overnight at 4°C. CD31 was visualised by a secondary antibody linked to Permanent Red. The slides were dehydrated in an oven at 60°C for one hour before cover slips were mounted using a Dako Cover Slipper. Staining without the primary antibodies was used as a negative control. Normal sinusoids in the adjacent liver tissue served as an internal positive control. 

### 2.5. Histological Evaluation

#### 2.5.1. Growth Pattern

The growth pattern was evaluated at the tumour periphery in the liver metastases as described previously [12, 20]. The growth pattern had to be present in more than 75% of the interface in order to be considered as a single growth pattern. If two growth patterns were present in the same metastasis and each growth pattern was represented in more than 25% of the visualised invasive front, the growth pattern was categorised as a mixed pattern, corresponding to the classification initially described by Vermeulen et al. [12]. When indicated, the growth pattern indicated first (e.g., P/D) was the one with the largest component at the invading front.

#### 2.5.2. uPAR-Immunoreactivity

The uPAR pAb has previously been validated by Illemann et al. [14]. In the present study, the sections stained for uPAR were evaluated by three independent observers (RE, MI, and ODL). Neutrophil granulocytes served as positive control for uPAR expression [22]. uPAR-immunoreactivity was scored at the invasive front (0.5 mm at the tumour periphery). The staining was observed both in budding cancer cells, macrophages, and myofibroblasts, which were identified in parallel sections by the immunoperoxidase staining for epithelial cells (pan-cytokeratin) and macrophages (CD68). The percentages of uPAR-positive cells were grouped into the following categories: 0, no uPAR-positive cells detected; 1, less than 5% positively stained cells; 2, between 5 and 10% positive cells; 3, 10–30% positive; 4, more than 30% positive cells. This method was modified from the method used by Laerum et al. for uPAR scoring in upper and lower gastric cancers [23, 24]. To simplify the uPAR score, the accessory cells positive for uPAR (myofibroblasts and macrophages) were assessed collectively instead of giving myofibroblasts and macrophages separate uPAR scores. 

#### 2.5.3. Angiogenesis

For the assessment of angiogenesis, the proliferation fractions of both endothelial cells (ECPs) and tumour cells (TCPs) were measured. ECP was obtained by counting approximately 200 normal endothelial cells (CD31-positive endothelial cells) and identifying proliferating endothelial cells (double positivity for Ki67 and CD31) at the invading front. By counting of 200 endothelial cells along the invading front, with about 20 rows of hepatocytes on one side and about 20 rows of cancer cells at the other side, the fraction of ECP could be determined. The counting was done manually with a denominator counting machine (http://www.denominatorcompany.com). In 18 of 62 liver metastases, the number of endothelial cells was between 168 and 200, because a total of 200 counted endothelial cells could not be reached. In 9 of 62 liver metastases, the number of endothelial cells was from 92–157. The TCP fraction was determined as the number of Ki67-positive cancer cells present among 200 cancer cells at the tumour periphery, in the areas of the highest expression of Ki67 (hot spots). The calculated ECP/TCP fraction was used as a morphometrical correlate of angiogenesis-dependent growth. Both ECP and TCP were assessed at a high magnification (x400).

### 2.6. Statistical Analysis

Kappa statistics was applied to assess the inter-observer agreement of the growth patterns and the uPAR score (weighted kappa). Comparisons of uPAR scores between growth patterns were done using the Kruskal-Wallis test and pairwise comparisons were then performed between the relevant pairs (D versus P, D versus R, P versus R). Data on angiogenesis was analysed using a general linear model with log transformed data. *P* values less than 5% were considered significant. Ninety-five percent confidence intervals are presented where applicable. All calculations were done using SAS (v9.2, SAS Institute, Cary, NC, USA). 

## 3. Results

### 3.1. Liver Metastases Growth Pattern

**Figure 2:**
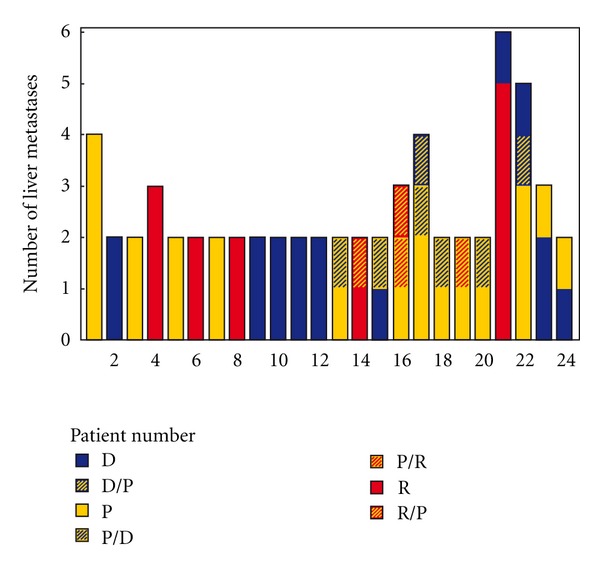
Column diagram of the growth pattern of the liver metastases. In 20 out of 24 patients, the same histological growth pattern was found in all metastases arising in the same patient, however, there was a second growth pattern component in one or two metastases from 8 of these patients. In 4 out of 24 patients the liver metastases were with different growth patterns, but in none of these patients more than two growth patterns were observed. Patient number 1 has 4 metastases, all with a pushing growth pattern. Patient number 2 has 2 metastases with a desmoplastic growth pattern. Patient number 13 has 2 metastases both with a pushing growth pattern, but the second metastasis has a component with a desmoplastic growth pattern (P/D). Patient number 21 has 6 metastases, 5 with a replacement growth pattern, and 1 with a desmoplastic growth pattern.

All 62 liver metastases were stained for reticulin fibres and haematoxylin and eosin in order to determine the growth pattern [12, 20]. Representative samples were also stained with Gomori trichrome. Seventy percent of the metastases were seen in the right lobe, 13% in the middle (segment 4), and 16% in the left lobe (Table 1). The growth pattern of each liver metastasis was determined after blinded evaluation by 4 independent observers (RE, MI, GVdE, PV). The inter-observation kappa among the four observers was between 0.52 and 0.69. For 18 out of 62 discrepant cases, a consensus was reached: typically there was a discrepancy in cases where one observer scored the metastasis with a certain growth pattern, while the other observer scored it with a mixed growth pattern. Of the total number of liver metastases analysed, 16 (25.8%) were desmoplastic, 21 (33.9%) were pushing, 13 (21%) were replacement type, and 12 (19.3%) were of a mixed type. In 20 out of 24 patients, the same histological growth pattern was found in all metastases arising in the same patient; however, there was a second growth pattern component in one or two metastases from 8 of these patients. In 4 out of 24 patients, the liver metastases were with different growth patterns, but in none of these patients more than two growth patterns were observed (Figures 1, 2 and Table 1). For the 20 out of 24 patients with the same growth pattern in all their metastases, the metastases were primarily found in different liver segments (Table 1). 

### 3.2. Expression of uPAR

**Figure 3:**
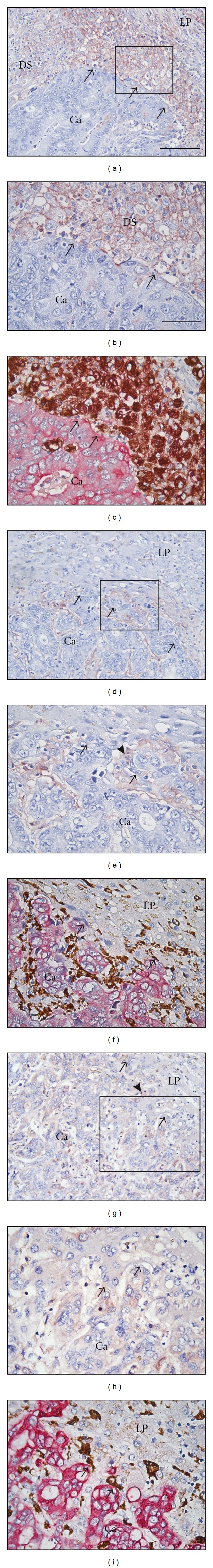
Localisation of uPAR and CD68-positive macrophages in colorectal liver metastases. Adjacent sections from a liver metastasis with desmoplastic growth (a)–(c), a liver metastasis with pushing growth pattern (d)–(f), and a liver metastasis with replacement growth pattern (g)–(i) were stained for uPAR (a), (b), (d), (e), (g), (h) or double stained for cytokeratin and CD68 (c), (f), (i). The uPAR-immunoreactivity was visualised by NovaRed, cytokeratin with Permanent Red, and CD68 with DAB. In the liver metastasis with desmoplastic growth pattern, strong expression of uPAR is seen in macrophages located at the invasive front within the desmoplastic zone (a), (b). Large numbers of macrophages were found at the front of the metastasis (c). In the liver metastasis with pushing growth pattern, uPAR expression is seen in a few macrophages and fibroblast-like cells intermingled with the tumour cells (d), (e). Few macrophages are located at the metastasis/liver parenchyma interface (f). In the liver metastasis with replacement growth pattern, uPAR expression is confined to some macrophages and fibroblast-like cells located between the tumour cells (g)-(h). Few macrophages are located at the metastasis/liver parenchyma interface (i). Black arrows points at the tumour periphery. Black arrowhead in (e) and (g) points at a uPAR-positive neutrophil, which is an internal positive control. Bar a: 100 *μ*m. Bar b: 50 *μ*m.

uPAR immunoperoxidase staining was performed on all liver metastases biopsies included in this study and listed in Table 4. In liver metastases with desmoplastic growth pattern, strong uPAR-immunoreactivity was detected at the invasive front primarily in macrophages but also in myofibroblasts and in some budding cancer cells close to the tumour periphery (Figures 3(a)-3(b)). In the liver metastases with a pushing growth pattern, few uPAR-positive cells were found at the invasive front (Figures 3(d)-3(e)). Scattered uPAR-positive cells were mainly macrophages observed both at the invasive front and in the centre of the metastasis. A few uPAR-positive fibroblast-like cells were also observed at the invading front. None of the cancer cells at the metastasis/liver parenchyma interface were uPAR-positive. In liver metastases with a replacement growth pattern, uPAR was found in a few macrophages and fibroblast-like cells at the invading front in a pattern much similar to that found in liver metastases with pushing growth pattern (see Figures 3(g)-3(h)). Macrophages and tumour cells were stained to illustrate the macrophage infiltration at the invasive front in the different growth patterns (Figures 3(c), 3(f), and 3(i)).

In general, positive uPAR-immunoreactivity was demonstrated in the neutrophils scattered throughout the tissue (black arrowhead in Figures 3(e)-3(g)). uPAR-positive macrophages were also located in luminal parts of the tumour glands as well as in central parts of the metastasis and often associated with necrosis (data not shown). In addition, staining for uPAR was observed in nerve bundles in few of the biopsies as well as in inflammatory cells located in inflammatory portal tracts (data not shown). 

The uPAR-immunoreactivity was scored in each of the samples as described in Section 2. For liver metastases with desmoplastic growth pattern, 14 metastases had an uPAR score of 3-4 at the invasive front, and 2 metastases had a score of 0–2. The median score was 4. For the liver metastases with pushing growth pattern, 9 out of 21 had an uPAR score of 3-4, while 12 had a score between 0–2. The median value was found to be 2. For replacement growth pattern, the uPAR expression at the invasive front was 3-4 for 11 out of 13 metastases, while the score 2 was given to 2 out of 13 metastases (median 3). For 12 out of the 62 CRLM with a mixed growth pattern, 7 metastases had score 0–2 and 5 metastases had score 3-4 (see Table 4). The weighed kappa values among the three observers were from 0.29–0.61 (paired comparisons).

The difference in uPAR-immunoreactivity at the invasive front was significant for the three growth patterns (*P* = 0.0013). Expression of uPAR was up-regulated at the invasive front of metastases with desmoplastic growth pattern in comparison to those with pushing growth pattern (*P* = 0.0008). When comparing the expression of uPAR in CRLM with replacement and pushing growth pattern, those with replacement had a more pronounced uPAR expression in comparison to those with pushing growth pattern (*P* = 0.0056). The difference in uPAR expression between metastases with desmoplastic and replacement growth pattern was non-significant (*P* = 0.26). 

### 3.3. Angiogenesis

**Figure 4:**
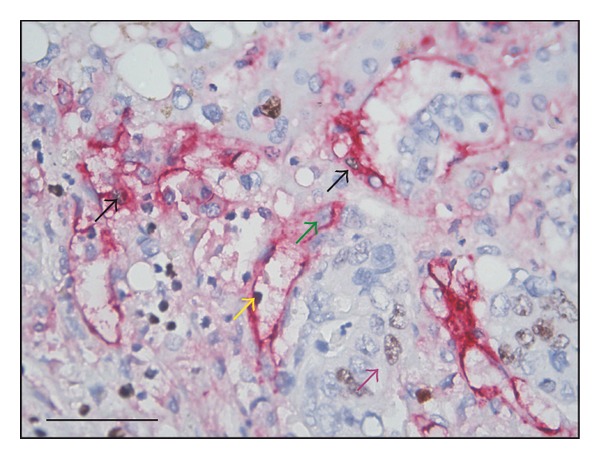
Proliferating endothelial cells. A section of a liver metastasis with pushing growth pattern was stained to identify proliferating endothelial cells using Ki67 and CD31. Ki67 was visualised by DAB, and CD31 by Permanent Red. Proliferating endothelial cells (black arrows) and non-proliferative endothelial cell (green arrow). A proliferative cancer cell is indicated with a purple arrow. Bar: 50 *μ*m.

**Figure 5:**
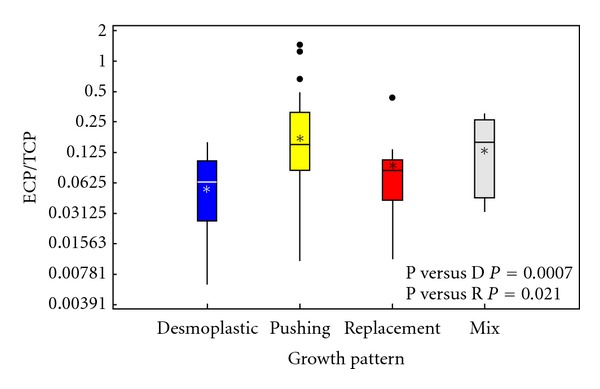
Box plot of ECP/TCP for each of the growth patterns. The boxes represent the median values of ECP/TCP (the horizontal line of each box) with the first quartile (lowest line) and the third quartile (upper line) for each of the growth patterns, D: desmoplastic, P: pushing, R: replacement, and Mix: mixed. The asterisks represent the geometric mean values. The vertical lines (whiskers) on both sides of the boxes represent 1.5 of the interquartile range at most. Outliers are indicated by dots.

For the assessment of angiogenesis-dependent growth, the mean fraction of ECP/TCP was calculated. For liver metastases with desmoplastic growth pattern, ECP/TCP was 0.048, for pushing growth pattern ECP/TCP was 0.160, and for replacement growth pattern, ECP/TCP was 0.068 (Figures 4 and 5). The estimates of the mean levels of ECP, TCP, and ECP/TCP (geometric means) are shown in Table 5. In a general linear model, the hypothesis of equal levels of ECP/TCP was rejected (*P* = 0.0046). The difference found between ECP/TCP in desmoplastic and pushing growth pattern was significant (*P* = 0.0007) with a mean of 0.30 (95% CI: 0.15–0.59). Also the difference between pushing and replacement growth patterns was significant, *P* = 0.021, with a mean of 2.33 (95% CI: 1.15–4.76). There was no difference between desmoplastic and replacement growth patterns (*P* = 0.36). These calculations were done on the 50 metastases where only one growth pattern was represented (Table 5 and Figure 5). 

ECP tended to be up-regulated in pushing growth pattern in comparison to desmoplastic and replacement growth pattern, but these findings were not significant (*P* = 0.38). TCP was up-regulated in the desmoplastic growth pattern in comparison to the pushing growth pattern (*P* = 0.0006) and there was a trend to a higher TCP in desmoplastic versus replacement growth pattern (*P* = 0.091) and between pushing and replacement growth pattern (*P* = 0.12). There was no correlation between ECP and TCP (*P* = 0.49). ECP, TCP, and ECP/TCP values are listed in Table 5.

### 3.4. Primary Tumour Characteristics

The primary tumour characteristics, such as the extent of invasion, the status of margin of resection, perineural invasion, lymphatic and venous invasion, lymph node metastases, and tumour budding were obtained from the pathology report from each patient (Table 2). No correlations could be found between the primary tumour characteristics and the growth pattern of the liver metastases. 

## 4. Discussion

Invasion of colorectal cancer cells into distant organs, such as the liver, is generally accompanied by pronounced activation of the stromal microenvironment, leading to desmoplasia, inflammation, and neo-vascularisation. CRLMs have been categorised into three growth patterns, each of which has characteristic morphological features [12], expression of the components of the plasminogen activation system [14] and angiogenesis [13]. This study demonstrates that the growth patterns of multiple liver metastases within a single patient were uniform in half of the patients evaluated, while in the other half one of the growth patterns was found to be dominating. The existence of the different growth patterns is in contrast to primary CRC tumours, where a desmoplastic zone at the invading front is always observed [14]. 

The liver metastasis growth patterns are different from those in experimental liver metastasis models, where two patterns depending on the route of tumour cell inoculation were observed [18]. Paku and Lapis infused mouse carcinoma cell lines with low and high metastatic potential and demonstrated two vascular patterns in liver metastases [18]. The authors did not describe specific growth patterns, but they could demonstrate difference in vascularity depending on the entry site into the liver of the cancer cells. In another animal study of CRC, liver metastases with a high vascular density were reduced after infusion of exogenous endostatin, demonstrating anti-angiogenic effects only in tumours with pronounced vascularisation, that is, sinusoidal type [19].

Our study showed that a central component of the plasminogen activation system, uPAR, was significantly up-regulated in macrophages at the invasive front of desmoplastic CRLM. In contrast, only scattered uPAR-positive macrophages were observed in liver metastases of the pushing growth pattern. Our results corroborate earlier studies on liver metastases of desmoplastic and pushing growth patterns [14], and do also add new data on uPAR expression in liver metastases of the replacement growth pattern. Taken together, these results suggest that the plasminogen activation system plays a preferential role in the development of desmoplastic CRLM. The precise contribution of uPAR and uPA remains, however, to be identified.

In this study, metastases with a pushing growth pattern were found to have the highest ECP/TCP fraction—a measure of angiogenesis dependent growth [12]. This high ECP/TCP fraction suggests that these metastases are more angiogenesis dependent than those of the other growth patterns, indicating that the growth pattern is likely regulated by distinct underlying biological processes. Our findings are slightly different than those reported in a previous study [12], where a significant difference was found between the ECP in the 3 growth patterns. In the present study, elevated ECP was found for the pushing growth pattern, but it was not found to be significant in comparison to the desmoplastic or replacement growth pattern. There was a significant difference in TCP between the three growth patterns in our study, but this significant difference was not found in the study by Vermeulen et al. [12]. Vermeulen et al. analysed autopsy material in contrast to the present study, where recently hepatic resection specimens were analysed. This could reflect essential differences in duration of hypoxia prior to tissue harvesting. As a result, the liver tissue affecting the endothelial and tumour cell proliferation could be stressed, which could explain the minor difference in ECP/TCP between the two studies. An alternative explanation might be that TCP was assessed in hot spot areas in the present study, whereas Vermeulen et al. mention an average TCP level throughout the tumour tissue.

Growth patterns have been related to differences in vascular phenotypes in other tumour types. In renal cell carcinoma, which is known to be highly angiogenic, an evaluation of angiogenesis in lung metastases was performed [25]. Two distinct patterns of lung metastases were identified, that is, an angiogenic and a non-angiogenic pattern [25]. The study concluded that the non-angiogenic metastases were most likely resistant to anti-angiogenesis treatment. Another study demonstrated biological differences in primary nonsmall cell lung carcinoma (NSCLC) [26]. They found three different growth patterns of the primary lung tumours as well as survival differences among the three subtypes. The conclusion of this study was that a classification of growth patterns could reflect differences in the biology of this malignancy [26].

In a study of primary tumours of NSCLC and their matched brain metastases, it was demonstrated that the vascular phenotype of the brain metastases was different with a greater proliferation rate and vascular maturity than those in their matched primary tumours [27]. Vascular phenotypes have also been studied by looking at the vessel maturation [28]. Mature blood vessels are suggested to be less sensitive to the angiogenesis inhibitor bevacizumab than immature vessels [29], and it was, therefore, suggested that bevacizumab had a decreased efficacy in patients diagnosed with NSCLC with brain metastases [27]. 

In gastric cancer, three distinct subtypes have been defined, based on histopathological and anatomical criteria [30], each subtype being associated with a unique epidemiology [30]. For metastatic CRC, the three growth patterns observed could represent differences in the underlying biology. A difference in survival has been demonstrated, with the desmoplastic growth pattern representing the group with the best outcome after a 2-year follow-up [20]. 

Halama et al. analysed the response to chemotherapy by evaluating immune responses in CRLM [31]. In this study, CD3^+^, CD4^+^, and CD8^+^ immune cells were analysed at the invasive front of the metastases. A high density of immune cells at the invasive front predicted a better effect of chemotherapy and longer progression-free survival. The authors described three different immune maps in the liver metastases, but the three distinct growth patterns of CRLM were not considered [31]. A linkage between immune maps and the growth pattern of the metastases would be very interesting to investigate in future studies.

Several phase II and III studies have shown that the combination treatment 5-FU, oxaliplatin, and bevacizumab, as well as the combination treatment with irinotecan, 5-FU, and bevacizumab were more effective than 5-FU, and oxaliplatin alone [5, 32, 33]. No biomarker is, however, presently available that could predict the response of the metastasis to bevacizumab treatment. The only validated marker for metastatic CRC is *Kras,* used for the selection of patients for treatment with the biological inhibitor cetuximab [34]. Microsatelite instability is a marker for different mutation phenotypes [35] and it is also a marker of better prognosis in metastatic CRC [36, 37] as well as a marker for the response to adjuvant therapy with 5-FU [38]. Whether the predominance of growth pattern in CRLM is linked to different phenotypes of metastatic CRC is still to be elucidated. Our investigations could, however, lead to a subclassification of metastatic CRC, offering a useful tool in the individualisation of oncological therapy. 

The “seed and soil” theory, where a tumour embolus is found in a recipient organ, was first described by Fuchs in 1882 [39] and thereafter commented by Paget in 1889 [40]. This theory is raised when the same growth patterns are observed in several liver segments. Similar growth patterns may be due to the same general reactions in the liver (soil) based on tumour cells (seed) derived from the same primary tumour. The organ microenvironment then determines which cells are recruited to the organ. On the other hand, tumour cells arriving to the liver may attract different host cells, such as macrophages, which modify further growth. That each patient has an individual pattern of metastasis would favour the view that biological properties in the primary tumour may be the primary factor. In this study, we have not been able to demonstrate correlations between primary tumour characteristics and the growth pattern of the liver metastases. Such correlations would be very interesting to study in a larger patient population.

It remains to be determined whether the growth pattern and vascularity of CRLM are determined by tumour or host specific factors or if it is a consequence of the route of tumour cell entry into the liver. The present study does, however, support earlier findings that linked the metastatic growth pattern to differences in the vascular activity of the CRLM at the invasive front [12, 20]. 

## 5. Conclusion

This is to our knowledge the first study of liver metastasis growth patterns in chemonaive patients resected for multiple CRLM. We show that liver metastases from patients resected for multiple CRLM have a tendency toward a uniform growth pattern. This suggests that the growth patterns are not a random phenomenon, but they could represent a selection of metastatic cells restricted to the prevailing growth patterns. Metastases with pushing growth pattern are characterised by a high angiogenic activity, while those with desmoplastic growth pattern have an up-regulated proteolytic activity.

Further studies are needed to elucidate if the three different growth patterns represent distinct subtypes of the primary tumour. If indeed liver metastases growth patterns could be predicted based on molecular biomarkers present in the primary tumours and each of the growth patterns could be associated to a distinct underlying biology, this could have major implications for the stratification of patients for oncological treatment. 

## Figures and Tables

**Table 1:** Patient characteristics. Colorectal liver metastases.

Patient number	Age	Gender	Number of liver metastases	Growth pattern	Liver segments
1	66	Male	4	P, P, P, P	3, 6, 7, 8
2	60	Female	2	D, D	4, 5
3	50	Male	2	P, P	3, 5 + 6
4	58	Male	3	R, R, R	2, 6, 8
5	55	Male	2	P, P	5, 7
6	43	Male	2	R, R	6, 7
7	60	Male	2	P, P	5, 5
8	43	Female	2	R, R	4, 8
9	64	Female	2	D, D	2, 7
10	56	Male	2	D, D	2, 5
11	62	Male	2	D, D	4, 7
12	62	Male	2	D, D	2, RL
13	77	Male	2	P, P/D	RL
14	74	Female	2	R, R/P	6, RL
15	60	Male	2	D, P/D	4B, 8
16	55	Male	3	P, P/R, R/P	RL
17	69	Female	4	P, P, P/D, D/P	2, 3, 6, 8
18	63	Male	2	R, R	6, 7
19	64	Female	2	P, P/R	RL
20	82	Female	2	P, P/D	2 + 3, 7
21	53	Male	6	R, R, R, R, R, D	3, 4B, 6, 7, 8, 8
22	75	Male	5	P, P, P, D/P, D	2, RL
23	57	Male	3	D, D, P	4, 5, 8
24	69	Female	2	D, P	2 + 3, 8

D: desmoplastic, P: pushing, R: replacement, P/D: pushing/desmoplastic, D/P: desmoplastic/pushing, R/P: replacement/pushing, P/R: pushing/replacement. The liver segments 1,2,3,4 are located in the left lobe (LL), while the liver segments 5,6,7,8 are located in the right lobe (RL).

**Table 2:** Patient characteristics. Colorectal primary tumour.

P#	TNMV stage	Diff grade	Resection margin	Perineural growth	Venous invasion	Number of lymph nodes	Macro Mets Lymph nodes	Micro Mets Lymph nodes	Budding Cancer Cells	Tumor site
1	T3N2M0V0	M	Free	No	No	14	6	0	UK	R
2	T2N0M1V1	M	Free	No	Yes	8	0	0	No	C
3	T3N1M1V1	M	Free	No	Yes	10	1	0	No	R
4	T3N2M1V1	M	Free	Yes	Yes	12	10	0	No	R
5	T3N1M0V0	M	Free	No	No	34	2	0	No	R
6	T3N2M0V0	L	Free	No	Yes	15	5	0	No	C
7	T3N1M1V0	M	Free	No	No	12	1	0	No	C
8	T4N2M1V1	M	Free	Yes	Yes	19	5	0	Yes	C
9	T3N0M1V0	M	Free	No	No	20	0	0	Yes	R
10	T3N1M0V0	M	Free	No	No	14	1	0	No	R
11	T3N1M1V2	M	Free	No	Yes	48	2	0	No	R
12	T3N0M0V0	M	Free	No	No	25	0	0	No	R
13	T4N1M1V0	H	Free	No	No	17	1	0	No	C
14	T3N1M1V0	M	Free	Yes	No	8	1	1	No	R
15	T3N2M1V2	L	Free	No	Yes	19	13	0	No	R
16	T4N2M1V1	M	Free	No	Yes	23	2	0	No	R
17	T3N0M0V0	L	Free	No	No	30	0	0	UK	C
18	T3N2M1V1	M	Free	Yes	Yes	16	9	0	No	R
19	T4N1M1V1	M	Free	Yes	Yes	18	2	1	Yes	R
20	T4N1M0V1	L	Free	Yes	Yes	44	1	0	No	C
21	T3N0M1V1	H	Free	Yes	Yes	18	0	0	No	C
22	T3N2M1V1	M	Free	Yes	Yes	41	12	0	No	R
23	T3N2M1V1	M	Free	No	Yes	33	15	5	No	C
24	T3N1M1V0	H	Free	No	No	12	3	0	UK	R

Based on the pathology reports from each patient, this table lists the TNMV-stage, differential grade, resection margin, perineural growth, venous invasion, number of resected lymph nodes, number of macrometastases in the resected lymph nodes, number of micrometastases of the resected lymph nodes, budding cancer cells and tumour site.

The differential grade was graded as H: high, M: moderate or L: low. The tumour site was either R: rectum or C: colon. UK: unknown.

**Table 3:** Primary antibodies.

Antigen	Clone	Cell marker	Source	Retrieval	Dilution
uPAR	Polyclonal	—	Finsenlab	Prot. K	1 *μ*g/ml
				15^′^37^°^C	
CD31	JC70A	Endothelial cells	Dako	TEG	1: 200
				20^′^98^°^C	
CK-pan	AE1/AE3	Epithelial cells, Cancer cells	Dako	Prot. K	1: 300
				15^′^37^°^C	
CK20	K_2_20.8	Epithelial cells, Cancer cells	Dako	Prot. K	1: 300
				15^′^37^°^C	
CD68	PG-M1	Monocytes, macrophages	Dako	Prot. K	1: 100
				15^′^37^°^C	
Ki 67	MIB-1	Proliferation marker	Dako	TEG	1: 50
				20^′^98^°^C	

**Table 4:** uPAR expression of the colorectal liver metastases.

Growth pattern	Number of CRLM	uPAR score, 0–2	uPAR score, 3-4	Median uPAR score
Desmoplastic	16	2	14	4
Pushing	21	12	9	2
Replacement	13	2	11	3
Mixed	12	7	5	2

**Table 5:** Endothelial cell proliferation (ECP)/tumour cell proliferation (TCP).

Growth pattern	Number of CRLM	ECP mean: 95% CI interval	TCP mean: 95% CI interval	ECP/TCP mean: 95% CI interval
Desmoplastic	16	0.020: 95% 0.013–0.040	0.50: 95% 0.28–0.89	0.048: 95% 0.029–0.079
Pushing	21	0.028: 95% 0.020–0.040	0.16: 95% 0.10–0.26	0.160: 95% 0.093–0.270
Replacement	13	0.019: 95% 0.011–0.030	0.27: 95% 0.20–0.38	0.068: 95% 0.039–0.120
Mixed	12	0.020: 95% 0.012–0.036	0.16: 95% 0.11–0.29	0.113: 95% 0.066–0.196

ECP: endothelial cell proliferation, TCP: tumour cell proliferation and ECP/TCP which is the value representing angiogenesis dependent growth.

ECP/TCP, pushing versus desmoplastic, *P* = 0.0007.

ECP/TCP, pushing versus replacement, *P* = 0.021.

## Abbreviations

CRC: Colorectal cancer
CRLM: Colorectal liver metastases
DAB: 3, 3′-diaminobenzidine
ECP: Endothelial cell proliferation
EGFR: Epidermal growth factor receptor
5-FU: 5-fluorouracil
mAb: Monoclonal antibody
NSCLC: nonsmall cell lung cancer
pAb: Polyclonal antibody
PAI-1: Plasminogen activator inhibitor-1
TCP: Tumour cell proliferation
uPA: Urokinase-type plasminogen activator
uPAR: uPA receptor
VEGF: Vascular endothelial growth factor
